# Highly active ozonides selected against drug resistant malaria

**DOI:** 10.1590/0074-02760160077

**Published:** 2016-05-30

**Authors:** Lis Lobo, Bruno de Sousa, Lília Cabral, Maria LS Cristiano, Fátima Nogueira

**Affiliations:** 1 Universidade Nova de Lisboa, Unidade de Ensino e Investigação de Parasitologia Médica, Global Health and Tropical Medicine, Lisboa, Portugal; 2 Universidade de Coimbra, Faculdade de Psicologia e Ciências de Educação, Coimbra, Portugal; 3 Universidade do Algarve, Centro de Ciências do Mar e Departamento de Química e Farmácia, Faro, Portugal

**Keywords:** malaria, drug resistance, ozonides

## Abstract

Ever increasing multi-drug resistance by *Plasmodium falciparum* is creating new challenges in malaria chemotherapy. In the absence of licensed vaccines, treatment and prevention of malaria is heavily dependent on drugs. Potency, range of activity, safety, low cost and ease of administration are crucial issues in the design and formulation of antimalarials. We have tested three synthetic ozonides NAC89, LC50 and LCD67 in vitro and in vivo against multidrug resistant *Plasmodium*. In vitro, LC50 was at least 10 times more efficient inhibiting *P. falciparum* multidrug resistant Dd2 strain than chloroquine and mefloquine and as efficient as artemisinin (ART), artesunate and dihydroartemisinin. All three ozonides showed high efficacy in clearing parasitaemia in mice, caused by multi-drug resistant *Plasmodium chabaudi* strains, by subcutaneous administration, demonstrating high efficacy in vivo against ART and artesunate resistant parasites.

Artemisinin (ART) and derivatives (ARTs) are the sole approved drugs effective against multidrug-resistant parasites (White 2008). ARTs are part of the artemisinin combination therapy (ACT’s) protocols, currently recommended by WHO as the first-line treatment for uncomplicated *Plasmodium falciparum* malaria (WHO 2006, Morris et al. 2010).

However, in addition to the recently detected resistance to ARTs by *P. falciparum* in Southeast Asia (Dondorp et al. 2009, Noedl et al. 2010), ARTs suffer from several liabilities that limit their therapeutic potential namely, cost, chemical stability, poor bioavailability and limiting pharmacokinetics (Avery et al. 1992, Ridley 2002, Gautam et al. 2009) and recrudescence (White 1999, Navaratnam et al. 2000).

The pharmacophore of ARTs is an endoperoxide bridge also present in trioxolanes (ozonides). Several synthetic trioxolanes have been tested for antimalarial activity and demonstrated in vivo activity against susceptible *Plasmodium berghei* ANKA and in vitro against *P. falciparum* strains (O’Neill et al. 2010).

The aim of the present study was to evaluate the efficacy of three of these compounds, LC50 (Vennerstrom et al. 2004, Dong et al. 2006), LCD67 (Vennerstrom et al. 2002) and NAC89 (Vennerstrom et al. 2004, Tang et al. 2007) (Supplementary figure 1 ) in vitro against the chloroquine (CQ) and mefloquine (MEF)-resistant *P. falciparum* strain Dd2 (Oduola et al. 1987) and in vivo against a multidrug-resistant strain of *Plasmodium chabaudi* (Rosario 1981), AS-ART (Hunt et al. 2010). AS-ART was derived from a CQ-resistant *P. chabaudi* (AS-30CQ) strain under ART pressure (Afonso et al. 2006).

## MATERIALS AND METHODS

*Synthesis of compounds tested* - Details on materials, methods and reaction conditions used for the preparation of the trioxolanes tested, NAC89, LC50 and LCD67 are provided as Supplementary data.

*In vitro cytotoxicity assay to human cells* - HepG2 - A16 human hepatic cell line viability was determined based on the methyl thiazol tetrazolium method (MTT) (Andrade-Neto et al. 2008). Briefly, an in vitro culture of HepG2 cells was maintained in standard culture conditions (37ºC, 5% CO_2_). Cells were seeded in a flat-bottomed 96-well tissue culture plate at a density of 1 × 104 cells/well and allowed to adhere overnight. After removing the medium, 200 μL of fresh medium, containing seven 2-fold dilutions (100000 ng/mL-0.01 ng/mL) of each compound, were added, and a negative control was prepared by adding 200 μL of drug free medium. The plate was incubated for 24 h under standard culture conditions. The medium was then substituted by fresh medium containing identical concentrations of the compounds, and the plates were incubated for another 24 h. At the end of the incubation period (48 h), 20 μL of 3-(4,5-dimethylthiazol-2-yl)-2,5- diphenyl-2H-tetrazolium [MTT; 5 mg/mL in phosphate buffered saline (PBS)] was added to each well, wells were incubated for 3 h at standard culture conditions, supernatant was removed, and 200 μL of acidified 2-propanol was added to each well. Absorbance was read at 570 nm, to produce a log dose-dependence curve. The LD_50_ was estimated for each compound by nonlinear interpolation of the dose dependence curve using GraphPad Prism 5.

*In vitro antimalarial activity* - *P. falciparum* clone Dd2 was cultured in human erythrocytes and synchronised twice with D-sorbitol before experiments, as described previously (Trager & Jensen 1976). Synchronised parasite cultures at ring stage (with 5% haematocrit and 1% parasitaemia) were treated for 48 h with serial dilutions of NAC89, LC50, LCD67, ART and DHA (10 ng/mL-0,156 ng/mL). After this incubation period, thin blood smears were prepared from control and treated wells, fixed with methanol, and Giemsa stained (20% Giemsa in buffered water for 20 min). To estimate the IC_50_, the parasitaemia determined for each concentration was normalised relative to control wells (no drug added) and plotted against the logarithm of the different drug dilutions. Corresponding IC_50_ values were estimated from dose-response curves generated using GraphPad Prism 5.

Selectivity index (SI) for each compound was determined by the ratio between HepG2 LD_50_ and *P. falciparum* IC_50_.

*In vivo antimalarial activity* - In vivo activity of NAC89, LC50 and LCD67, against *P. chabaudi* strains AS-30CQ (sensitive to ART) and AS-ART (resistant to ART) selected for ARTs resistance elsewhere (Hunt et al. 2010), was assessed by treating infected mice subcutaneously with two different doses (10 and 50 mg/kg/day, or 2 mg/kg/day for NAC89) of each ozonide, once daily, for three consecutive days. Groups of five females Balb/C mice, per compound and dose, including PBS as negative control. ART was used as a positive control, since the profile of antimalarial activity in strains AS-30CQ and AS-ART was previously known. Parasitaemia was monitored in tail blood smears prepared daily, fixed with methanol and stained with Giemsa, starting at day 4 after first treatment, until day 20. The area under the curve (AUC) was estimated using GraphPad Prism 5, based on the daily-recorded parasitaemias.

Animals were handled and cared according to the internationally accepted laboratory animals’ use, care and welfare guidelines, in use at IHMT. IHMT’s vivarium is licensed by the Portuguese Food and Veterinary Directorate General for the breeding and use of animals (http://www.ihmt.unl.pt/en/vivarium/).

## RESULTS AND DISCUSSION

*In vitro antimalarial activity* - The antimalarial effect of NAC89, LC50 and LCD67 (Supplementary figure 1 ) against *P. falciparum* blood-stage forms of the CQ and mefloquine (MEF) resistant strain Dd2 was evaluated. The most active of the compounds was LC50, with an IC_50_ = 0.8 ± 0.2 ng/mL, followed by NAC89 and LCD67 (Table).

**TABLE:** In vitro evaluation of cytotoxicity in HepG2 and activity of new ozonides against chloroquine and mefloquine resistant *Plasmodium falciparum* and in vivo activity against artemisinin resistant *Plasmodium chabaudi* strains in three different doses

In vitro				In vivo					
Compound	*P. falciparum* (Dd2)	Cell line HepG2	SI	(*P. chabaudi*) AS-30CQ (mg/kg)			(*P. chabaudi*) AS-ART (mg/kg)		
	
	IC_50_ (ng/mL)	LD_50_ (ng/mL)		2	10	50	2	10	50

				AUC (mean ± sem)			AUC (mean ± sem)		
PBS	-	-	-	111.8 ± 15.6			99.6 ± 33.7		
LC50	0.8 ± 0.2	31841.0	39801.2	-	7.6 ± 2.1	0	-	58.0 ± 16.5	6.7 ± 2.0
LCD67	2.0 ± 0.1	23576.0	11788.0	-	109.9 ± 18.9	15.2 ± 4.1	-	40.5 ±17.5	29.2 ± 2.9
NAC89	1.7 ± 0.2	53818.0	31657.6	29.6 ± 7.6	7.9 ± 4.5	-	32.0 ± 7,9	8.3 ± 2.7	-
ART	0.7 ± 0.1	-	-	-	51.8 ± 14.5	11.9 ± 4.5		89.6 ± 16.4	24.7 ± 3.8
DHA	1.1 ± 0.2	-	-	-	-	-	-	-	-

ART: artemisinin; AUC: area under the curve; CQ: chloroquine; DHA: dihidroartemisinin; IC50: inhibitory concentration for 50% of the parasites; LD50: lethal dose to 50% of the cells; PBS: phosphate buffered saline; SI: selective index.

The IC_50_ values obtained for Dd2 are in line with previous determinations of in vitro activity against the *P. falciparum* CQ-resistant strain K1 (IC_50_ = 62.0 ± 4.0 ng/mL to CQ and 3.0 ± 0.1 ng/mL to MEF) (Vennerstrom et al. 2004). When compared to antimalarials in use, LC50 was as good as ART and better than dihidroartemisinin (DHA) inhibiting quinoline-resistant *P. falciparum* blood-stages in vitro. LC50 was 77.5 times more efficient than CQ, 3.7 times more efficient than MEF, 1.4 times more efficient than DHA and as efficient as ART inhibiting quinoline-resistant parasites (Table). Compounds LCD67 and NAC89 also exhibited higher activity than CQ and MEF against the quinoline-resistant parasites (Dd2). The results indicate that LC50, LCD67 and NAC89 do not show cross-resistance with CQ or MEF, possibly acting through a different mechanism. In vitro cytotoxicity of the compounds NAC89, LC50 and LCD67 was determined using mammalian HepG2 cells and the colorimetric MTT. The resulting LD_50_s indicate that the compounds tested have very low cytotoxicity (Table).

The three compounds demonstrated exceptional growth-inhibitory activities against multi-drug resistance *P. falciparum* parasites (in the nanomolar range), while showing neglectable effect on the growth or viability of mammalian cells (SI ≥ 11000) (Table), this indicating an ample therapeutic window.

In vivo antimalarial activity of LC50, LCD67 and NAC89 - Even though NAC89 exhibited the second best SI (still very high), at the dose of 50 mg/kg/day two mice exhibited a small lesion at the injection site, which was not observed with the dose of 10 mg/kg/day. Therefore, to be able to test for dose response effect, a second dose of 2 mg/kg/day was used. It is currently accepted that the phenotype of ART drug resistance is reflected by the increased parasitaemia clearance time. Additionally, *P. chabaudi* strains with an AS genetic background are not lethal, mice resolve infection in ~ 20 days without treatment (Bagnaresi et al. 2009). Mean of all measurements, height of peak, time to reach peak and time to return to baseline level are the usual parameters to express the efficacy of compounds (Matthews et al. 1990). By definition, the AUC is a summary calculation used when serial measurements on each subject under study are carried out. We propose the AUC to express the evolution of the parasitaemia after treatment, as a more biologically relevant estimator, because it incorporates both the magnitude and the duration of parasitaemia patency. Thus, parasitaemia evolution after treatment was monitored and expressed as AUC (Table). AUCs reflecting antimalarial activity of control drug (ART) against both AS-30CQ and AS-ART *P. chabaudi* clones were calculated (Supplementary figure 2 ). The AUC value for the ART- sensitive strain (AS-30CQ) was significantly lower than the AUC for the resistant one (AS-ART) as compared by the two-sided Mann-Whitney test (p < 0.001).

When compared to the untreated control (PBS with 10% DMSO), ART produced a significant reduction in parasitaemia and a dose response effect. The AUC values reflected the expected phenotype described elsewhere (Hunt et al. 2010).

In both resistant and sensitive strains, when compared to the untreated control, LC50 and NAC89 induced a significant reduction of the AUC (p < 0.001) at both doses, while the LCD67 only caused a significant reduction at a dose of 50 mg/kg (p < 0.001). Considering the three trioxolanes and ART, NAC89 was the most active against the AS-ART since even at a dose as low as 10 mg/kg the AUCs were significantly lower (p < 0.01) (Supplementary figure 3 , Table).

When compared to equivalent doses of the control drug ART, all three tested ozonides followed the same pattern, reducing 1.5 -11.0 times the correspondent AUC. When compared with ART, the LC50 compound showed better antimalarial activity at both doses, against both (AS-30CQ and AS-ART) strains. At the highest dose (50 mg/kg), LC50 reduced the parasitaemia 3.7 and 11.9 times on the resistant and sensitive strains, respectively. At the lowest concentration (10 mg/kg), the reduction was 1.5 and 6.8 times, respectively. LC50 had already demonstrated in vivo activity against *P. berghei* ANKA (drug susceptible strain) when administrated subcutaneously in a dose of 100 mg/kg (survival up to 30 days) and orally (100 mg/kg, survival up to 10.7 days) (Vennerstrom et al. 2004).

In conclusion, the compounds tested showed an excellent antimalarial activity in vitro and in vivo against resistant strains. Although NAC89 needs further toxicity evaluations, the other ozonides LC50 and LCD67 exhibited an excellent performance in vitro, against a multidrug resistant *P. falciparum* strain, and in vivo, against a CQ-resistant and ART-resistant rodent malaria strains. The high SI of all three compounds against *Plasmodium* parasites compared to mammalian cells, as well as their potent in vivo activity against resistant strains of rodent malaria parasites (*P. chabaudi*), suggest that this class of compounds deserves further investigation.
