# Supplementary material for: Highly active ozonides selected against drug resistant malaria
Source: Mem Inst Oswaldo Cruz. 2016 May 30;111(7):450–3. doi: 10.1590/0074-02760160077 (PMC4957497; doi:10.1590/0074-02760160077)
Supplement: Supplementary file 1 [file 0074-0276-mioc-0074-02760160077-sd.pdf]

# Synthesis of compounds tested

*Preparation of trioxolanes* - The synthetic approach followed to trioxolanes is depicted in Schemes 1-2. Synthetic procedures for each compound prepared are also provided in this section.

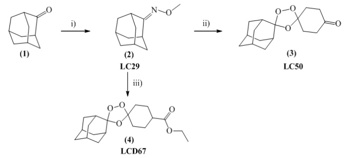

Scheme 1: synthetic route to trioxolanes LC50 and LC67. Reagents and conditions: (i) Pyridine, $MeONH_2$, MeOH; (ii) 1-4: cyclohexane, $O_3$, DCM/pentane, -78ºC; (iii) Ethyl 4-oxocyclohexanecarboxylate, $O_3$, DCM/Pentane, -78ºC.

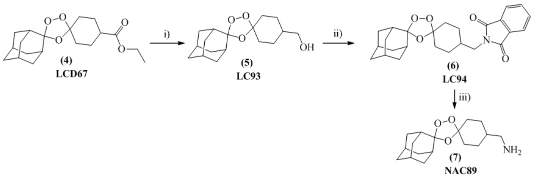

Scheme 2: synthetic route to trioxolane NAC89 from LC67. Reagents and conditions: (i) $LiBH_4$, $Et_2O$, $LiBH(Et)_3$, r.t.; (ii) Phthalimide, $Ph_3P$, DIAD, THF, 0ºC; (iii) Hydrazine hydrate, chloroform/MeOH, 60ºC.

*Preparation of O-methyl-2-adamantanone oxime (LC29)*

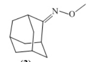

(2)
LC29

To a solution of 2-adamantanone (4.51 g, 30 mmol) in methanol (30 mL) were added pyridine (4.5 mL, 55.6 mmol) and methoxylamine hydrochloride (3.76 g, 45.0 mmol). The reaction mixture was stirred at room temperature for 48 h. The final mixture was concentrated and then diluted with DCM (50 mL) and water (50 mL). The organic layer was separated and the aqueous layer was extracted with DCM (30 mL). The combined organic extracts were washed with aqueous HCl (1 M; 30 mL x2), then with saturated aqueous NaCl (30 mL). The final organic extract was dried over $MgSO_4$, filtered and concentrated under reduced pressure to give *O*-methyl-2-adamantanone oxime (4.77 g, 26.6 mmol, 89%) as a colorless solid (m.p. 69-70ºC). $^1H$ NMR (400 MHz, $CDCl_3$): δ 1.78-1.97 (12H, m), 2.53 (1H, s), 3.45 (1H, s), 3.81 (3H, s); MS (MALDI-TOF): m/z 180.02 $[M]^+$.

*Synthesis of 1, 2, 4-trioxolanes - General procedure*: Trioxolanes were prepared by coupling O-methyl-2-adamantanone oxime (2) with a cyclohexanone derivative, through ozonolysis.

Ozone, produced with an ozone generator Sander Labor-Ozonizator 301.7 (0.5 L/min $O_2$, 140 V), was passed through a solution of dichloromethane at -78ºC and flushed into a solution of O-methyl ketone oxime and a ketone, in pentane/dichloromethane (6:4) at 0ºC. After completion, the solution was flushed with nitrogen for 5 min and concentrated under reduced pressure at room temperature to give a crude material that was purified by column chromatography.

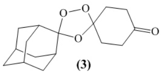

(3)
LC50

*Adamantane-2-spiro-3'-8'-oxo-1', 2', 4'-trioxaspiro[4,5]decane (LC50).*

A solution of O-methyl 2-adamantanone oxime (1.5 g, 8.4 mmol) and 1,4-cyclohexanedione (1.9 g, 11 mmol) in pentane (60 mL) and dichloromethane (40 mL) was treated with ozone (as described in general procedure I). The crude product was purified by column chromatography (silica gel; ethyl acetate/n-hexane 1/9) to give product **LC50** (1.18 g, 3.52 mmol, 42%) as a colorless solid (m.p. 127-128ºC). $^1$H-NMR ($CDCl_3$): δ 1.69-2.02 (m, 14H), 2.14 (t, J = 6.9 Hz, 4H), 2.51 (t, J = 7.0 Hz, 4H); $^{13}$C NMR (100 MHz, $CDCl_3$): 25.9, 26.31, 31.09, 32.59, 34.25, 35.70, 36.18, 37.35, 106.46, 111.95, 208.90; MS (EI), m/z 278.9 $[M]^+$.

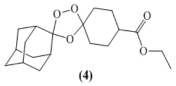

(4)
LCD67

*Adamantane-2-spiro-3'-8'-ethoxycarbonyl-1', 2', 4'-trioxaspiro[4,5]decane (LCD67).*

A solution of O-methyl 2-adamantanone oxime (3.58 g, 20 mmol) and ethyl 4-oxocyclohexanecarboxylate (3.40 g, 20 mmol), in pentane (60 mL) and DCM (40 mL), was treated with ozone, according to the previous procedure. The crude product was purified by column chromatography (silica gel, ethyl acetate/n-hexane 1/9) to afford trioxolane **LCD67** as a colorless oil (3.10 g, 9.2 mmol, 46%). $^1$H-NMR ($CDCl_3$): δ 1.26 (3H, t, J=6.9Hz), 1.70-1.76 (11H, m), 1.92-2.03 (12H, m), 2.33 (1H, m), 4.15 (2H, dd, J=7.2Hz, J=14.3Hz); MS (MALDI-TOF), m/z 337.34 $[M]^+$.

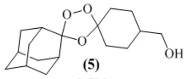

(5)
LC93

*Adamantane-2-spiro-3'-8'-hydroxymethyl-1', 2', 4'-trioxaspiro[4,5]decane (LC93).*

A solution of **LCD67** (3.8 g, 11.3 mmol), lithium borohydride (5.7 mL, 11.3 mmol, 2M in THF) and lithium triethylborohydride (1.15 mL, 1.13 mmol, 1M in THF) in ether (15 mL) was stirred overnight, at room temperature. The reaction mixture was diluted with ether (5 mL), washed with aqueous NaOH (3M; 2 x 10 mL), brine and water (2 x 10 mL). The organic extract was dried over $MgSO_4$, filtered, and concentrated under reduced pressure to give product **LC93** (3 g, 10.2 mmol, 90%) as a colorless solid (m.p. 99-101ºC). $^1$H-NMR ($CDCl_3$): δ 1.25 (2H, m), 1.51-2.08 (21H, m), 3.46 (2H, t, J=4.8Hz); MS (MALDI-TOF), m/z 318.30 $[M+Na]^+$.

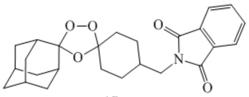

(6)
LC94

*Adamantane-2-spiro-3'-8'-phtalimidomethyl-1', 2', 4'-trioxaspiro[4,5]decane (LC94).*

A solution of **LC93** (2.8 g, 9.52 mmol) in dry THF (25 mL) was cooled to 0ºC. $Ph_3P$ (3.5 g, 1.33 mmol), phthalimide (1.55 g, 10.5 mmol) and DIAD (2.6 mL, 1.33 mmol) were gradually added. The mixture was stirred at room temperature for 24 h. The solvent was then evaporated to dryness and the crude product was purified by column chromatography (silica gel, ethyl acetate/n-hexane 1/9) to give product **LC94** (3.22 g, 7.62 mmol, 80%) as a white powder (.mp. 149-151ºC). $^1$H NMR (300 MHz, $CDCl_3$): δ 1.30-1.34 (2H, m), 1.51-2.08 (21H, m), 3.55 (2H, d, J = 7.0Hz), 7.71 (2H, m), 7.84 (2H, m); MS (MALDI-TOF), m/z 462.19 $[M+K]^+$.

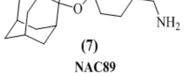

(7)
NAC89

*Adamantane-2-spiro-3'-8'-(aminomethyl)-1', 2', 4'-trioxaspiro[4,5]decane (NAC89).*

A solution of **LC94** (3.20 g, 7.56 mmol) and hydrazine monohydrate (1.45 g, 45.4 mmol) in chloroform and methanol (7:3, 50 mL total) was heated at 60ºC for 35 h. The reaction mixture was cooled to room temperature and filtered to remove solid by-products. The filtrate was washed with water (50 mL) and brine (50 mL), dried over $MgSO_4$, filtered, and concentrated to give product **NAC89** (1.72 g, 5.87 mmol, 77%) as light yellow oil. $^1$H NMR (400 MHz, $CDCl_3$): δ 1.14-1.33 (3H, m), 1.68-1.96 (22H, m), 2.54 (2H, d, J=6.4Hz); MS (MALDI-TOF), m/z 293.20 $[M]^+$.

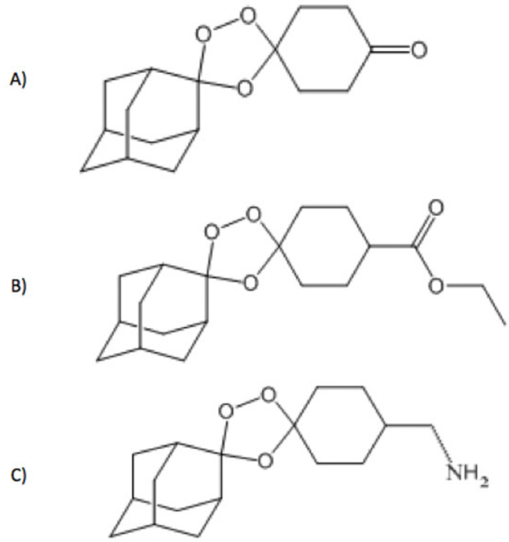


Fig. 1: chemical structure of the tested componds. (A) LC50, (B) LCD67 and (C) NAC89.

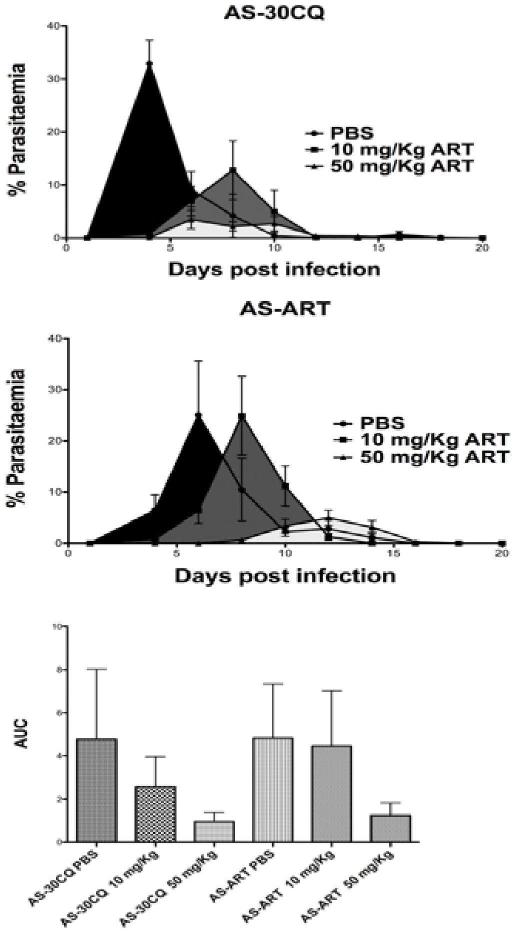


Fig. 2: parasitaemia evolution in mice infected with sensitive (AS-30CQ) and resistant (AS-ART) *Plasmodium chabaudi* strains treated with artemisinin, correlates with the correspondent AUC values. Parasitaemia was monitored daily. AUC corresponding to the parasitaemia evolution until reaching zero, it is presented as mean. Black-PBS; Gray - 10 mg/mL; White - 50 mg/mL ($p < 0.001$).

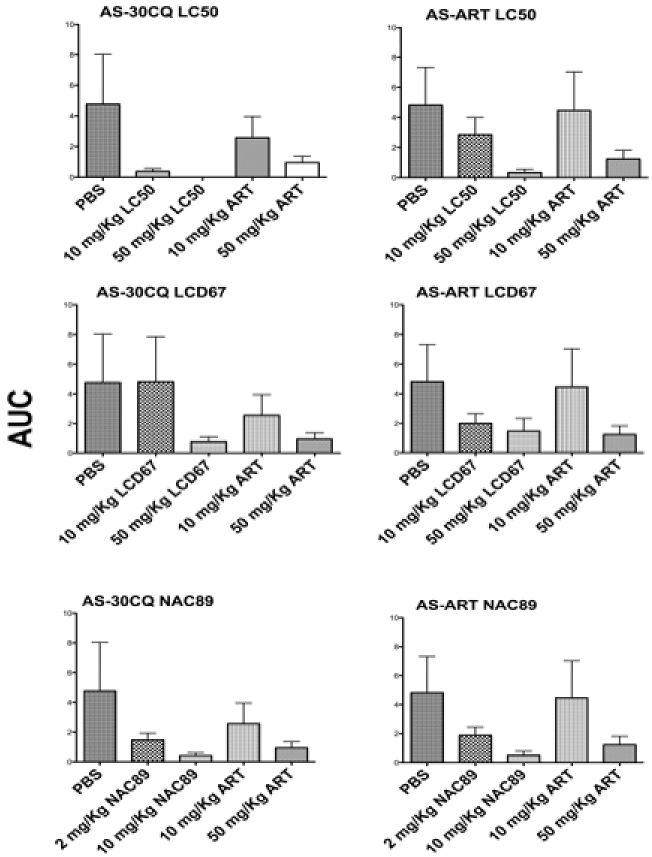


Fig. 3: in vivo activity of new ozonides against chloroquine and artemisinin resistant *Plasmodium chabaudi* strains. *P. chabaudi*-infected mice were treated once daily subcutaneously for three consecutive days. Parasitaemia was monitored daily, starting at day five after first treatment and expressed as the AUC corresponding to the parasitaemia evolution until reaching zero, it is presented as mean ± SEM ($p < 0.001$).
